# Neurobehavioral risk factors influence prevalence and severity of hazardous substance use in youth at genetic and clinical high risk for psychosis

**DOI:** 10.3389/fpsyt.2023.1143315

**Published:** 2023-04-20

**Authors:** Carolyn M. Amir, Simon Kapler, Gil D. Hoftman, Leila Kushan, Jamie Zinberg, Kristin S. Cadenhead, Leda Kennedy, Barbara A. Cornblatt, Matcheri Keshavan, Daniel H. Mathalon, Diana O. Perkins, William Stone, Ming T. Tsuang, Elaine F. Walker, Scott W. Woods, Tyrone D. Cannon, Jean Addington, Carrie E. Bearden

**Affiliations:** ^1^Department of Psychiatry and Biobehavioral Sciences, Semel Institute for Neuroscience and Human Behavior, University of California, Los Angeles (UCLA), Los Angeles, CA, United States; ^2^Department of Psychiatry, University of California, San Diego (UCSD), San Diego, CA, United States; ^3^Department of Psychiatry, Zucker Hillside Hospital, Long Island, NY, United States; ^4^Department of Psychiatry, Harvard Medical School at Beth Israel Deaconess Medical Center and Massachusetts Mental Health Center, Boston, MA, United States; ^5^Department of Psychiatry, San Francisco Veterans Affairs (SFVA) Medical Center, University of California, San Francisco (UCSF), San Francisco, CA, United States; ^6^Department of Psychiatry, University of North Carolina, Chapel Hill, NC, United States; ^7^Institute of Genomic Medicine, University of California, San Diego, La Jolla, CA, United States; ^8^Departments of Psychology and Psychiatry, Emory University, Atlanta, GA, United States; ^9^Department of Psychiatry, Yale University, New Haven, CT, United States; ^10^Department of Psychology, Yale University, New Haven, CT, United States; ^11^Department of Psychiatry, Hotchkiss Brain Institute, University of Calgary, Calgary, AB, Canada; ^12^Department of Psychology, University of California, Los Angeles (UCLA), Los Angeles, CA, United States

**Keywords:** 22q11.2 deletion syndrome, clinical high risk for psychosis (CHR), psychosis, substance use, cannabis

## Abstract

**Background:**

Elevated rates of alcohol, tobacco, and cannabis use are observed in both patients with psychotic disorders and individuals at clinical high risk for psychosis (CHR-P), and strong genetic associations exist between substance use disorders and schizophrenia. While individuals with 22q11.2 deletion syndrome (22qDel) are at increased genetic risk for psychosis, initial evidence suggests that they have strikingly low rates of substance use. In the current study, we aimed to directly compare substance use patterns and their neurobehavioral correlates in genetic and clinical high-risk cohorts.

**Methods:**

Data on substance use frequency and severity, clinical symptoms, and neurobehavioral measures were collected at baseline and at 12-month follow-up visits in two prospective longitudinal cohorts: participants included 89 22qDel carriers and 65 age and sex-matched typically developing (TD) controls (40.67% male, M_age_ = 19.26 ± 7.84 years) and 1,288 CHR-P youth and 371 matched TD controls from the North American Prodrome Longitudinal Study-2 and 3 (55.74% male; M_age_ = 18.71 ± 4.27 years). Data were analyzed both cross-sectionally and longitudinally using linear mixed effects models.

**Results:**

Controlling for age, sex, and site, CHR-P individuals had significantly elevated rates of tobacco, alcohol, and cannabis use relative to TD controls, whereas 22qDel had significantly lower rates. Increased substance use in CHR-P individuals was associated with increased psychosis symptom severity, dysphoric mood, social functioning, and IQ, while higher social anhedonia was associated with lower substance use across all domains at baseline. These patterns persisted when we investigated these relationships longitudinally over one-year. CHR-P youth exhibited significantly increased positive psychosis symptoms, dysphoric mood, social functioning, social anhedonia, and IQ compared to 22qDel carriers, and lower rates of autism spectrum disorder (ASD) compared to 22qDel carriers, both at baseline and at 1 year follow-up.

**Conclusion:**

Individuals at genetic and CHR-P have strikingly different patterns of substance use. Factors such as increased neurodevelopmental symptoms (lower IQ, higher rates of ASD) and poorer social functioning in 22qDel may help explain this distinction from substance use patterns observed in CHR-P individuals.

## 1. Introduction

Excessive substance use occurs at elevated rates in patients with psychotic disorders (1–8) and cannabis use in particular has been suggested to play a role in the onset of psychosis (9–17). Patients with schizophrenia are 4.6 times more likely to use and abuse substances than the general population (18). Similar rates of hazardous substance use, ranging from 22% to over 50%, are reported for individuals at clinical high risk for psychosis (CHR-P)—individuals experiencing attenuated psychotic-like experiences preceding the onset of psychosis (3, 19)—compared to typically developing controls (TD) (3, 20–23). In contrast, a notable lack of substance use has been reported in people with chromosome 22q11.2 deletion syndrome (22qDel) (24, 25). This copy number variant (CNV) confers one of the strongest genetic risk factors for psychosis (24–30).

The reasons for the substantially elevated rates of hazardous substance use in individuals with idiopathic psychotic disorders are not fully understood but are thought to be linked to the brain’s dopaminergic pathways, associated with the reward properties of drugs and positive symptoms of psychosis (4, 31). Certain genetic factors lead to an increased risk of substance use and psychotic disorders, and genetic risk is thought to interact with environmental factors including the social environment (4, 10, 32, 33). That individuals with 22qDel may need little or no environmental insult to develop psychosis is consistent with the liability threshold model; stronger genetic predisposition may ‘tip the scales’ toward phenotypic expression of psychosis, even in the absence of environmental risk factors (34–37).

While not yet elucidated in 22qDel, neurobehavioral traits associated with hazardous substance use have been studied in CHR-P. Positive symptom severity including rates of unusual thought content and suspiciousness is greater in CHR-P who use illicit substances compared to CHR-P non-users (19, 38, 39). CHR-P and individuals with first-episode psychosis report social engagement as a primary reason for cannabis use (40–42) and higher levels of social functioning are associated with elevated substance use rates in CHR-P (43). Mood enhancement is also cited by CHR-P as a primary motivation for substance use seeking (40), and increased depression symptoms are associated with elevated substance use rates in adolescence (44). CHR-P cannabis lifetime users have higher intelligence quotient (IQ) scores compared to non-users (45).

To our knowledge, this is the first study to directly compare substance use patterns in CHR-P and 22qDel, which offers a unique opportunity to contrast clinical and genetic factors underlying hazardous substance use during adolescent development. While CHR-P individuals are identified on the basis of early psychosis symptom presentation and behavioral risk factors, 22qDel offers a unique “genetics-first” approach to understanding psychosis spectrum disorders. Unlike CHR-P, 22qDel participants are identified based on a specific genetic risk factor, that is, a deletion at the 22q11.2 locus. Our age-matched comparison groups have similar rates of conversion to psychosis (46, 47), medication usage, and gender distribution, and medical exclusion criteria and therefore present a valuable opportunity to compare these two groups at high risk for development of psychosis. Investigating factors associated with decreased rates of substance use in 22qDel could point to a protective phenomenon, with differential implications for prediction and treatment/prevention in subsets of individuals at high risk for psychosis.

Here, we investigated rates of substance use in two prospective longitudinal cohorts, youth with CHR-P symptoms and youth with 22q11.2 deletion syndrome, as well as demographically-matched typically developing (TD) controls, with the hypothesis that CHR-P youth will have elevated rates of substance use relative to TD controls, both cross-sectionally and longitudinally over 1 year, whereas 22qDel participants will not. Second, we aimed to investigate the relationship of substance use to psychiatric symptoms and neurobehavioral traits. We predicted that better social functioning and higher IQ, as well as increased dysphoric mood and psychosis symptom severity would be associated with increased rates of substance use, while social anhedonia would be inversely related with substance use. We then examined differences in these measures between 22qDel carriers and CHR-P youth to elucidate the potential influence of social functioning, social anhedonia, dysphoric mood, psychosis symptom severity, and IQ on substance use patterns in this population.

## 2. Materials and methods

### 2.1. Study procedures and participants

This study examined substance use over time in both 22qDel individuals with molecularly confirmed 22q11.2 deletions (*n* = 89, 45% male) and age and sex-matched TD controls (*n* = 65, 46.15% male) and in CHR-P youth and age and sex-matched typically developing controls. CHR-P participants were recruited as part of the North American Prodromal Longitudinal Studies 2 (NAPLS 2) (48) and NAPLS 3 (49), designed to investigate predictors and mechanisms of transition to overt psychosis. This paper reports on 737 CHR-P youth (57.53% male) and 275 TD control subjects (50.18% male) who completed substance use assessments. Use of the NAPLS 3 cohort in this study is intended to replicate our NAPLS 2 baseline findings in a population at higher risk for conversion to psychosis (at “enhanced” risk; 40% likelihood of psychosis conversion) (49, 50) to test whether findings hold in an enhanced sample. NAPLS 3 participants include 551 “enhanced” CHR-P participants (56.99% male) and 96 TD control subjects (50% male). Detailed descriptions of the recruitment procedures and measures are reported elsewhere (48, 49).

Individuals with 22qDel included in this study represent a subset of participants ascertained as part of an ongoing longitudinal study at the University of California, Los Angeles. Details of the recruitment and methods for this study are described elsewhere (51). Age and sex-matched TD participants were recruited from local communities via web-based advertisements and flyers/brochures in local schools, pediatric clinics, and other community sites. Exclusion criteria for all study participants included significant neurological or medical conditions (unrelated to 22qDel) that might affect brain structure or function, history of head injury with loss of consciousness, and insufficient fluency in English.

CHR individuals and TD controls were recruited for NAPLS studies, which were approved by the Institutional Review Boards of all eight NAPLS sites. Written informed consent, including parental consent, was obtained from all adult participants and parents/guardians of minors. The University of California, Los Angeles Institutional Review Board approved all study procedures and informed consent documents related to 22q11.2 CNV studies. Participants under the age of 18 years provided written assent, while their parent or guardian completed written consent in each study. Across both cohorts, we restricted our analyses to participants age 12 and older. As maximal data were available for the first two timepoints of the studies, longitudinal analyses included baseline visits and one-year follow-up visits.

### 2.2. Clinical status

A summary of measures and timepoints analyzed in this study are presented in Supplementary Table S1. See Supplementary material for full details on clinical measures used to assess clinical status.

#### 2.2.1. Substance use

Substance use in NAPLS 2 and NAPLS 3 participants was assessed using the Alcohol Use Scale/Drug Use Scale (AUS/DUS) (52) at all visits. Both severity (1 = abstinent, 2 = use without impairment, 3 = abuse, 4 = dependence) and frequency of substance use (0 = no use, 1 = once or twice per month, 2 = 3–4 times per month, 3 = 1–2 times per week, 4 = 3–4 times per week, 5 = almost daily) were collected for tobacco, alcohol, cannabis, cocaine, opiates, phencyclidine (PCP), amphetamines, methylenedioxy-methylamphetamine (MDMA), γ-hydroxybutyric acid (GHB), huffing (inhaling glue, other volatiles), hallucinogens, and other drugs. Frequency of tobacco use was the only item rated differently (0 = no use, 1 = occasionally, 2 = less than 10 times per day, 3 = 11–25 per day, 4 = more than 25 per day). Longitudinal group comparisons were conducted for NAPLS 2 data; baseline results were analyzed to test for replication in NAPLS 3, as control participants did not complete the AUS/DUS at follow-up in NAPLS 3. 22qDel and respective TD control participants were assessed at each timepoint via SCID interview for substance use/abuse within the past 6 months, and the Child Behavior Checklist (CBCL; 53) was used to assess lifetime substance use or abuse, including alcohol, non-prescription drug use, and tobacco use. The CBCL was completed by parents of participants aged 12–18.

#### 2.2.2. Social functioning

The Global Functioning: Social scale was used to measure social functioning in both cohorts. Social functioning is calculated by two metrics: (a) current degree of social functioning (“GFS current”); and (b) highest level of social functioning in the past year (“GFS highest”). Developmentally appropriate, detailed descriptions are provided to illustrate the range of functioning captured by each point on the scale, with lower scores indicating more impairment (53).

### 2.3. Statistical analyses

Analyses were conducted in R 4.1.2 using statistical software package lme4 (54). We compared individuals with 22qDel to respective age and sex-matched TD controls, and CHR-P youth to a separate age and sex-matched TD control group (with TD controls as the reference groups). For NAPLS 2 and 22qDel cohorts, timepoints included intake visit and one-year follow-up visits, and longitudinal models included a group-by-time interaction. The use of substances other than alcohol, tobacco or cannabis was either minimal or absent in all samples; therefore, only alcohol, tobacco, and cannabis were considered for further statistical analysis. To determine whether CHR-P and 22qDel show different rates of substance use relative to TD controls, linear mixed models with substance use as the dependent variable (DV) and participant group as the independent variable (IV) were used to test for group differences in substance use (with controls as the reference group). We tested replication of NAPLS 2 results with NAPLS 3 baseline data, as substance use information was not collected longitudinally in NAPLS 3. Age, sex, and site were included covariates, and participant ID was included as the random effects term. FDR correction (55) was applied within each model on effects of subject group.

To test relationships between substance use and clinical symptoms, linear mixed models with each symptom domain as DV and substance use characterization as IV were tested for control and CHR-P participants at baseline in NAPLS 2 and NAPLS 3. In NAPLS 2, we tested relationships between baseline clinical symptoms and substance use at follow-up in CHR-P, additionally controlling for baseline substance use rates. Main effects of each model were FDR-corrected for multiple comparisons.

We then tested differences in clinical symptom measures between 22qDel participants and CHR-P to elucidate neurobehavioral traits influencing substance use patterns in this population (with 22qDel carriers as the reference group). Linear mixed models with substance use as the DV and participant group as the IV were used to test for differences in clinical symptom measures at baseline and longitudinally with participant age, sex, and site included as covariates. FDR correction was applied within each model on effects of subject group.

## 3. Results

Demographic variables at baseline are presented for each group in Tables 1, 2. There were significant differences in medication use between 22qDel carriers and TD controls, as well as between CHR-P and TD controls, such that TD controls had lower rates of medication use. In the CHR-P group there were significantly higher rates of Hispanic TD control participants compared to the 22qDel group and significantly higher rates of non-white participants. At one-year follow-up, 9.93% of CHR-P participants and 13.73% of 22qDel carriers met criteria for conversion to a psychotic disorder.

**Table 1:** Baseline characteristics: 22qDel carriers and TD controls.

Baseline	Typically developing control subjects	22q11.2 deletion carriers
N, total	61	89
Age, years (SD), age range^b^	17.18 (4.28); 12–28	20.17 (10.18); 12–61
Males, n (%)	29 (47.5%)	40 (45%)
Non-white, n (%)^a^	22 (39.3%)	8 (8.99%)
Hispanic^a^	17 (27.87%)	14 (15.73%)
Psychosis n (%)^b^	0 (0.00%)	12 (13.48%)
ASD, n (%)^b^	0 (0.00%)	37 (41.57%)
Anxiety disorder (%)	0 (0.00%)	0 (0.00%)
Alcohol use (%)^a^	10 (16.39%)	3 (3.37%)
Substance use (non-prescription) (%)	2 (3.28%)	2 (2.24%)
Tobacco use	2 (3.28%)	1 (1.12%)
Medication, n (%)^b^	2 (3.28%)	41 (46.07%)
Anti-psychotics	0 (0%)	15 (16.85%)
*Antidepressants/Mood stabilizers	0 (0%)	13 (14.6%)
Stimulants	1 (1.54%)	5 (5.62%)
Other medication	1 (1.54%)	8 (9.00%)
No medication	57 (93.44%)	48 (53.93%)

^a^Control > 22qDel (*p* < 0.05). ^b^22qDel > Control (*p* < 0.05). *Antidepressants and mood stabilizers were recorded together in the 22qDel study.

**Table 2:** Baseline characteristics: NAPLS 2 and 3 (CHR-P subjects and TD controls).

NAPLS 2 baseline	TD controls	CHR-P
N, total	275	737
Age, years (SD), age range^a^	19.76 (4.68); 12–34	18.49 (4.24); 12–35
Males, n (%)	138 (50.18%)	424 (57.53%)
Non-white, n (%)	125 (45.45%)	310 (42.06%)
Hispanic	48 (17.45%)	138 (18.72%)
Anxiety disorder (%)^a^	0 (0.00%)	171 (23.61%)
Medication, n (%)^a^	7 (2.55%)	296 (40.16%)
Anti-psychotics	0 (0%)	132 (17.91%)
Mood stabilizers	0 (0%)	26 (3.53%)
Stimulants	3 (1.09%)	52 (7.06%)
Antidepressants	2(0.73%)	191 (25.92%)
Other medication	4 (1.45%)	86 (11.67%)
No medication	262 (95.27%)	441 (59.84%)
NAPLS 3 baseline	TD controls	Clinical high risk for psychosis—enhanced participants
N, total	96	551
Age, years (SD), age range	18.60 (4.22); 12–30	18.42 (4.04); 12–30
Males, n (%)	48 (50%)	314 (56.99%)
Non-white, n (%)^b^	51 (53.13%)	244 (44.28%)
Hispanic	24 (24.00%)	125 (22.69%)
Anxiety disorder(%)^a^	0 (0.00%)	150 (27.72%)
Medication, n (%)^a^	1 (1.04%)	256 (46.46%)
Anti-psychotics	0 (0.0%)	114 (20.69%)
Mood stabilizers	0 (0.0%)	21 (3.81%)
Antidepressants	0 (0.0%)	175 (31.76%)
Stimulants	1 (1.04%)	40 (7.26%)
Other medication	0 (0.0%)	81 (14.7%)
No medication	0 (0.0%)	294 (53.36%)

^a^CHR-P > Control (*p* < 0.05). ^b^Control > CHR-P (*p* < 0.05).

### 3.1. Substance use at baseline and over time

Results of mixed models revealed that CHR-P had greater frequency and severity of both cannabis and tobacco use compared with controls both at baseline and longitudinally (see Supplementary Tables S2, S3 for full results). There was no statistical difference in alcohol use severity between controls and CHR-P (Supplementary Table S3). All group differences in baseline substance use observed in NAPLS 2 replicated in NAPLS 3 (see Supplementary Table S4). Controlling for tobacco use in cannabis models and psychotropic medication use in all substance use models did not affect results.

No 22qDel participants endorsed substance abuse or dependence in the past 6 months at baseline or follow-up timepoints. 22qDel carriers endorsed significantly lower rates of alcohol use than controls at both baseline (*b =* −0.217, *q* = 0.007) and longitudinally (*b =* −0.336, *q* = 0.007), and lower rates of non-prescription drug use at one-year follow-up (*b =* −0.284, *q* = 0.007). Less than 4% of 22qDel carriers endorsed substance use at any timepoint, compared to 20% of TD controls endorsing substance use (see Table 1 for full results).

### 3.2. Relationships between psychosis symptom domains and substance use

Due to the lack of substance use in 22qDel participants, relationships between substance use and clinical symptoms could only be investigated in CHR-P (NAPLS 2 and NAPLS 3) participants (Tables 3, 4 and Supplementary Table S3). In both cohorts of CHR-P participants, positive symptoms were significantly positively associated only with cannabis use severity and frequency, and baseline positive symptom severity was positively associated with cannabis use severity but not frequency at follow-up in CHR-P participants (Table 4).

**Table 3:** Results of cross-sectional models in NAPLS 2.

Cross-sectional	Effect of substance use: control subjects		Effect of substance use: CHR-P subjects	
Positive symptoms	*β*	*q*-value	*β*	*q*-value
Alcohol use frequency	0.041	0.615	0.044	0.126
Alcohol use severity	−0.015	0.739	0.018	0.299
Cannabis use frequency	0.129	0.169	**0.096**	**0.011**
Cannabis use severity	0.113	0.187	**0.105**	**0.010**
Tobacco use frequency	0.106	0.226	0.026	0.251
Tobacco use severity	0.052	0.583	0.064	0.067
*Dysphoric mood*				
Alcohol use frequency	0.074	0.071	0.028	0.065
Alcohol use severity	0.046	0.133	**0.068**	**0.009**
Cannabis use frequency	**0.211**	**<0.001**	**0.071**	**0.009**
Cannabis use severity	**0.125**	**0.030**	**0.083**	**0.006**
Tobacco use frequency	**0.112**	**0.035**	**0.073**	**0.009**
Tobacco use severity	0.060	0.107	**0.067**	**0.011**
*Social anhedonia*				
Alcohol use frequency	−0.075	0.324	**−0.139**	**<0.001**
Alcohol use severity	−0.051	0.324	**−0.121**	**<0.001**
Cannabis use frequency	−0.055	0.324	**−0.092**	**0.003**
Cannabis use severity	−0.056	0.324	**−0.116**	**<0.001**
Tobacco use frequency	0.014	0.521	**−0.127**	**<0.001**
Tobacco use severity	0.005	0.552	**−0.164**	**<0.001**
*IQ*				
Alcohol use frequency	0.131	0.080	**0.199**	**<0.001**
Alcohol use severity	0.109	0.127	**0.169**	**<0.001**
Cannabis use frequency	−0.030	0.648	**0.086**	**0.029**
Cannabis use severity	0.033	0.627	**0.102**	**0.010**
Tobacco use frequency	−0.018	0.689	0.016	0.427
Tobacco use severity	0.027	0.653	0.021	0.393
*GFS current*				
Alcohol use frequency	0.134	0.138	**0.253**	**<0.001**
Alcohol use severity	**0.178**	**0.040**	**0.232**	**<0.001**
Cannabis use frequency	0.017	0.701	**0.055**	**0.031**
Cannabis use severity	0.048	0.567	**0.101**	**0.001**
Tobacco use frequency	−0.065	0.469	**0.136**	**<0.001**
Tobacco use severity	−0.034	0.637	**0.173**	**<0.001**
*GFS highest*				
Alcohol use frequency	**0.187**	**0.014**	**0.178**	**<0.001**
Alcohol use severity	**0.199**	**0.013**	**0.173**	**<0.001**
Cannabis use frequency	0.026	0.603	0.035	0.106
Cannabis use severity	0.049	0.487	**0.066**	**0.026**
Tobacco use frequency	−0.040	0.545	**0.097**	**0.004**
Tobacco use severity	−0.019	0.637	**0.125**	**<0.001**

**Table 4:** Relationships between baseline neurobehavioral measures and substance use at one-year follow-up in NAPLS 2 CHR-P.

Positive symptoms	*β*	*q*-value
Alcohol use frequency	0.007	0.872
Alcohol use severity	0.016	0.715
Cannabis use frequency	0.054	0.262
Cannabis use severity	**0.095**	**0.046**
Tobacco use frequency	0.007	0.889
Tobacco use severity	−0.009	0.847
*IQ*		
Alcohol use frequency	**0.178**	**<0.001**
Alcohol use severity	**0.158**	**<0.001**
Cannabis use frequency	**0.118**	**0.019**
Cannabis use severity	**0.133**	**0.008**
Tobacco use frequency	−0.006	0.902
Tobacco use severity	0	0.995
*Dysphoric mood*		
Alcohol use frequency	0	0.998
Alcohol use severity	0	0.985
Cannabis use frequency	0	0.996
Cannabis use severity	0.027	0.579
Tobacco use frequency	**0.120**	**0.012**
Tobacco use severity	**0.113**	**0.019**
*Social anhedonia*		
Alcohol use frequency	**−0.166**	**<0.001**
Alcohol use severity	**−0.152**	**<0.001**
Cannabis use frequency	**−0.172**	**<0.001**
Cannabis use severity	**−0.185**	**<0.001**
Tobacco use frequency	**−0.221**	**<0.001**
Tobacco use severity	**−0.193**	**<0.001**
*Anxiety diagnosis*		
Alcohol use frequency	−0.009	0.843
Alcohol use severity	−0.033	0.486
Cannabis use frequency	−0.027	0.591
Cannabis use severity	−0.032	0.521
Tobacco use frequency	−0.009	0.856
Tobacco use severity	0.004	0.940
*GFS current*		
Alcohol use frequency	**0.098**	**<0.001**
Alcohol use severity	**0.111**	**<0.001**
Cannabis use frequency	**0.070**	**<0.001**
Cannabis use severity	**0.069**	**<0.001**
Tobacco use frequency	**0.067**	**<0.001**
Tobacco use severity	**0.058**	**<0.001**
*GFS highest*		
Alcohol use frequency	**0.145**	**<0.001**
Alcohol use severity	**0.109**	**0.009**
Cannabis use frequency	0.028	0.241
Cannabis use severity	0.032	0.222
Tobacco use frequency	**0.099**	**0.021**
Tobacco use severity	**0.102**	**0.020**

Bold values represent a *q*-value < 0.05, considered statistically significant.

Dysphoric mood at baseline in CHR-P in NAPLS 2 was positively associated with cannabis use frequency and severity, tobacco use frequency and severity, and alcohol use severity (Table 3). In NAPLS 2, social anhedonia was inversely associated with substance use across all domains in CHR-P at baseline. Further, social anhedonia at baseline in CHR-P was inversely associated with substance use across all domains at one-year follow up (Table 4). Baseline dysphoric mood in CHR-P in NAPLS 2 was positively associated with tobacco use frequency but no other substance use domain at follow-up. In NAPLS 3, dysphoric mood was positively associated with tobacco use frequency and severity in CHR-P and social anhedonia was inversely associated with substance use across all domains in CHR-P at baseline.

### 3.3. Relationship between social functioning and substance use

In NAPLS 2, alcohol use frequency and severity were both positively associated with social functioning in controls at baseline. In CHR-P, baseline social functioning was positively associated with substance use across all domains at baseline and was positively associated with all substance use domains at follow-up (Table 4). While the social functioning results in CHR-P replicated in NAPLS 3, alcohol use was not associated with social functioning in controls in NAPLS 3 (Supplementary Table S5).

### 3.4. 22qDel carriers vs. CHR-P: Group differences in neurobehavioral symptoms

The CHR-P cohort (NAPLS 2) exhibited increased total psychosis symptom severity, positive symptom severity, dysphoric mood and social anhedonia, as well as higher IQ scores compared to 22qDel carriers, both at baseline and longitudinally (Table 5). In contrast, 22qDel carriers showed lower global social functioning than CHR-P, both at baseline and longitudinally. 22qDel carriers exhibited significantly higher rates of ASD compared to CHR-P.

**Table 5:** Differences in neurobehavioral measures between 22qDel (reference group) and NAPLS CHR subject groups.

	Effect of subject group		Effect of time		Group * time interaction	
Cross-sectional	*β*	*q*-value	*β*	*p*-value	*β*	*p*-value
Total psychosis symptoms	**0.229**	**<0.001**				
Positive symptoms	**1.412**	**<0.001**	–	–	–	–
Dysphoric mood	**1.010**	**<0.001**	–	–	–	–
Social anhedonia	**0.357**	**<0.001**	–	–	–	–
SCID: ASD	**−2.171**	**<0.001**	–	–	–	–
GFS	0.029	0.156	–	–	–	–
GFS highest	**0.398**	**<0.001**	–	–	–	–
IQ	**1.521**	**<0.001**	–	–	–	–
*Longitudinal*						
Total psychosis symptoms	**0.381**	**<0.001**	0.017	0.904	**−0.502**	**0.002**
Positive symptoms	**0.623**	**<0.001**	−0.028	694	**−0.456**	**<0.001**
Dysphoric mood	**0.434**	**<0.001**	−0.038	0.647	**−0.340**	**0.002**
Social anhedonia	**0.183**	**0.026**	−0.012	0.886	−0.161	0.163
SCID: ASD	**−0.502**	**<0.001**	0.124	0.112	−0.120	0.220
GFS current	−0.025	0.433	0.010	0.242	0.061	0.587
GFS highest	**0.137**	**<0.001**	0.088	0.330	−0.064	0.582
IQ	**0.389**	**<0.001**	0.007	0.931	0.124	0.238

Bold values represent a *q*-value < 0.05, considered statistically significant.

## 4. Discussion

Our study represents, to our knowledge, the first direct comparison of substance use patterns and neurobehavioral correlates in youth at clinical and genetic high risk for psychosis. Specifically, we compared youth with 22q11.2 deletions (22qDel) to a clinically/behaviorally defined high-risk cohort (CHR-P youth) and found support for extremely low rates of substance use in 22qDel, but elevated rates of substance use in CHR-P youth, relative to TD controls. These results suggest that despite conferring elevated risk for psychosis (56), neurobehavioral factors related to the 22q11.2 deletion appear to be protective against initiating and/or continuing substance use. We then tested cross-sectional and longitudinal associations between substance use and neurobehavioral traits in CHR-P youth and found broadly that increased severity of positive psychosis symptoms and dysphoric mood, as well as better social functioning and higher IQ, were associated with greater substance use frequency and severity. In contrast, greater social anhedonia was associated with significantly lower substance use, across domains. These patterns persisted when we investigated these relationships longitudinally over one-year. Finally, we directly compared these neurobehavioral measures in 22qDel carriers and CHR-P youth and found that CHR-P youth exhibited significantly increased positive psychosis symptoms, dysphoric mood, social functioning, social anhedonia, and IQ compared to 22qDel carriers, but significantly lower rates of ASD compared to 22qDel carriers.

This work expands upon prior studies reporting elevated rates of substance use in individuals at high risk for psychosis (3, 20, 22, 23, 44) by examining associated neurobehavioral factors both cross-sectionally and longitudinally and in parallel with a population at genetically high risk for psychosis. Drugs of misuse directly or indirectly activate the mesolimbic dopamine pathway, which is associated with the reward properties of drugs and positive symptoms of schizophrenia (31). An array of dopaminergic abnormalities has been reported in CHR-P populations, including alterations in midbrain and striatal responses to reward assessed via functional neuroimaging studies, and elevated dopamine synthesis, storage, and release, observed in positron emission tomography and single photon emission computed tomography studies [for a review see (57)]. In addition, CHR-P individuals display impaired reward processing associated with abnormal striatal activity during task performance (58, 59), related to symptom severity. Research on 22qDel mesolimbic differences using neuroendocrine and peripheral dopaminergic markers has revealed dopaminergic abnormalities including disrupted dopaminergic neurotransmission (60), along with deficits in pleasure responses and reduced activation in medial frontal areas during reward anticipation (61, 62). Our study supports a body of work indicating that elevated rates of cannabis use in individuals at high risk for psychosis are related to positive psychosis symptom severity, which may be mediated by alterations in these reward-related pathways [for reviews, see (22, 63)], while there is mixed evidence on the association between alcohol or tobacco use and positive psychosis symptoms (3). By contrast, positive symptom severity was significantly lower in 22qDel carriers.

CHR-P youth displayed significantly better social functioning and reduced rates of ASD compared to 22qDel carriers, and social functioning was positively associated with substance use, both at baseline and longitudinally, within CHR-P. These results are consistent with previous descriptions of withdrawn behavior and problems with peer social interaction in 22qDel carriers (64), as well as with a previous single-site study finding higher levels of social functioning associated with elevated substance use rates in CHR-P (65). CHR-P individuals report social engagement as a primary reason for substance use (41–43). It has been hypothesized that social skills facilitate drug acquisition in individuals with psychotic disorders such that poorer social skills make illicit substances more difficult to obtain (66, 67), especially where there are legal barriers (68). While not yet studied in CHR-P, higher peer engagement was associated with increased risk for substance use particularly during adolescence (69–72), which has been theorized to be attributable to an increase in substance use accessibility with higher social engagement. In addition to high rates of ASD (73), social phobia was overrepresented in 22qDel (74). That social functioning was significantly lower in 22qDel carriers compared to CHR-P youth may represent a protective factor against hazardous substance use for 22qDel carriers.

The positive association between dysphoric mood and substance use domains in CHR-P is consistent with previous reports of CHR-P individuals citing mood enhancement as motivation for seeking cannabis (29, 41, 75). Significantly lower dysphoric mood in 22qDel carriers compared to CHR-P youth may indicate that while mood enhancement is a primary motivation for drug-seeking in CHR-P individuals, this motive may not be as strong for 22qDel carriers.

Social anhedonia in CHR-P was significantly inversely correlated with substance use at baseline and predicted decreased substance use at follow-up. This supports previous findings that CHR-P cannabis users have significantly lower levels of social anhedonia compared to CHR-P non-users; one possible explanation is that lower social anhedonia drives peer engagement and substance-seeking for social motives, increasing substance use and access (20, 41, 43). That levels of social anhedonia were lower in 22qDel carriers compared to CHR-P may indicate that lower rates of social anhedonia in 22qDel may not be driving peer engagement and substance use in the same way as for CHR-P individuals. Lower social functioning and elevated rates of ASD in 22qDel carriers may again serve as a protective barrier to acquisition and use. While a relationship between psychosis symptoms and cannabis use have been reported in NAPLS 3 (20), our work expands upon these findings by focusing specifically on the enhanced sample of participants and investigating alcohol and tobacco use. Substance use rates and relationships between neurobehavioral measures and substance use broadly replicated in our enhanced CHR-P sample (NAPLS 3), providing support that these findings hold in a population at especially high risk for psychosis.

Consistent with previous literature (46), we also found that the likelihood of substance use increased with higher IQ within the CHR-P group. IQ scores in CHR-P youth were, on average, significantly higher compared to those of 22qDel carriers. While we could not test the relationship between IQ and substance use in 22qDel patients given the minimal substance use reported in our cohort, a previous study reported substance use increased with higher IQ scores in 22qDel carriers (24). This prior work speculated as an explanation that individuals with more severe intellectual disability (ID) are more likely to be cared for or protected by systems providing supervision. Substance use rates are relatively low in individuals with idiopathic ID compared to TD individuals, similarly to 22qDel (76, 77). Some variables found to influence substance use in individuals with ID include social pressure and the desire to increase social inclusion and overcome loneliness (78–80). Future work should investigate whether 22qDel carriers who do have hazardous substance use patterns are undertreated. Further, the influence of environmental factors, such as peer usage and neighborhood factors, and the interplay between such environmental factors and behavior should be examined to determine their effects on substance use patterns in 22qDel. Such factors may moderate the observed rates of substance use in individuals at high risk for psychosis.

The current study has several important clinical implications. That 22qDel, despite conferring increased genetic risk for psychosis, is protective against problematic substance use offers insight into behavioral risk factors that can be targeted for intervention in individuals at CHR-P. Drugs of abuse can contribute to the positive symptoms of psychosis, and people with any substance use disorder have an earlier age of schizophrenia onset [for a review, see (81)]. Cannabis in particular can be a contributory risk factor for psychotic disorder, and animal models show that the developing brain is susceptible to cannabis-induced brain morphological and circuitry changes (82, 83). As cannabis legalization becomes more prevalent and access increases, it is becoming increasingly important to identify avenues for behavioral intervention for CHR-P.

Our findings suggest the importance of neurobehavioral risk factors and social context in influencing substance use, pointing to interventions that target social influence in disrupting the initiation and continuation of substance use. Future research should interrogate the clinical effectiveness of targeting peer influence on substance use patterns; for example, identifying whether an individual is at high-risk for hazardous substance use based on their social exposures. Clinicians may use information about social context to help inform their approach to intervention and potentially mitigate harmful substance use through identifying and addressing social factors including peer use and accessibility. These considerations may be of particular importance in geographical regions with more permissive cannabis legislation. Therefore, clinicians may take into account regional factors such as urbanicity, neighborhood, and legislative context in their assessments.

Strengths of this study include the large sample of individuals at CHR-P and the sample of participants with 22qDel, a relatively rare disorder with an estimated prevalence of one in 3,000–4,000 live births (84). Adolescence and young adulthood are crucial developmental periods for studying the relationship between substance use and psychosis [e.g., (34)]; our study provides longitudinal insight into the progression of clinical symptoms and behavioral trajectories during neurodevelopment, whereas many previous studies utilize cross-sectional designs. Previous literature has focused primarily on the relationship between cannabis use and schizophrenia rather than psychosis spectrum disorders, and mixed findings have been attributed to differences in the temporal relationship between onset of cannabis use and onset of psychosis (85). Most previous studies on substance use in psychiatric populations lack details on the severity and frequency of use, and often conflate types of substance use. Moreover, very few include a healthy comparison group.

### 4.1. Limitations

This study also has several important limitations. A majority of NAPLS data was collected pre-legalization of cannabis in the United States, and rates of substance use in this sample may become greater in regions where cannabis is legalized. Details on cannabis use including type of cannabis, dose, and whether cannabidiol was also consumed were not collected. Urine toxicology data would have also provided important biological information on cannabis use in the sample. In NAPLS 2 there were few “abuse” and “dependence” occurrences recorded for substance use, and this may limit our ability to detect a contribution of heavy substance use to psychotic transition and/or clinical outcome. Demands of the NAPLS studies may also have deterred heavier substance users from participating, which may have biased our samples. While psychotropic medication usage rates are comparable among CHR-P and 22qDel patients and controlling for medication use did not affect our results, other medical issues specific to 22qDel may also explain decreased substance use. Extremely low rates of substance use in 22qDel carriers rendered this study underpowered our ability to detect associations between substance use and neurobehavioral traits in 22qDel carriers.

### 4.2. Conclusion

In conclusion, we found that despite higher rates of substance use in CHR-P individuals compared with controls, individuals with 22qDel, although at increased risk for psychosis, had markedly lower prevalence of substance use relative to TD controls. As this is the first study to investigate neurobehavioral traits underlying substance use patterns in CHR-P compared to 22qDel carriers, more research is needed into other factors such as environmental risk that may interplay with genetic factors to confer this effect. 22qDel could be a valuable model to study factors underlying substance use in the general population.

## Data availability statement

Publicly available datasets were analyzed in this study. This data can be found at: https://nda.nih.gov/edit_collection.html?id=2275 (NAPLS 3 data accession number 2275) and https://nda.nih.gov/edit_collection.html?id=2414 (22q11.2 data accession number 2414) NAPLS 2 data will be made available upon reasonable request.

## Ethics statement

The studies involving human participants were reviewed and approved by the Institutional Review Board of the University of California, Los Angeles, the Institutional Review Board of Emory University, the Institutional Review Board of Harvard University, the Institutional Review Board of the University of Calgary in Canada, the Institutional Review Board of the University of California, San Diego, the Institutional Review Board of the University of North Carolina at Chapel Hill, the Institutional Review Board of Yale University, the Institutional Review Board of Zucker Hillside Hospital. Written informed consent to participate in this study was provided by the participants’ legal guardian/next of kin.

## Author contributions

CA designed the study analyses, conducted the statistical analyses, and drafted the manuscript. CB, LKu, and JZ contributed to 22q11.2 study design and data collection. LKu oversaw recruitment and participant assessment for 22q11.2 studies. JA, KC, BC, DM, DP, MT, MK, EW, SW, and TC were responsible for the design of the NAPLS studies and supervision of the data collection. SK, GH, and LKe provided critical input on the manuscript text and interpretation of results. All authors participated in manuscript revisions and have given final approval of this version for submission.

## Funding

This work was supported by the National Institute of Mental Health (grants U01MH081984, U01 MH081928, P50 MH080272 to JA, grants R01 MH60720, U01 MH082022, and K24 MH76191 to TC; grant U01MH082004-01A1 to DP, grant U01MH08202 to SW, grant UO1 MH081857-05 to BC; grants R01 MH085953, U01MH101779 to CB), NIH National Center for Advancing Translational Science (grant UL1TR001881 to GH), UCLA Friends of the Semel Institute Research Scholar Award (GH), Karen Seykora NARSAD Young Investigator Grant from the Brain and Behavior Research Foundation (GH), Harvey L. and Maud C. Sorensen Foundation Fellowship (GH), Burroughs Wellcome Fund Career Award for Medical Scientists (GH), and the Commonwealth of Massachusetts (grant SCDMH82101008006 to Seidman).

## Conflict of interest

The authors declare that the research was conducted in the absence of any commercial or financial relationships that could be construed as a potential conflict of interest.

## Publisher’s note

All claims expressed in this article are solely those of the authors and do not necessarily represent those of their affiliated organizations, or those of the publisher, the editors and the reviewers. Any product that may be evaluated in this article, or claim that may be made by its manufacturer, is not guaranteed or endorsed by the publisher.
